# Regulation of Nrf2/GPX4 Signaling Pathway by Hyperbaric Oxygen Protects Against Depressive Behavior and Cognitive Impairment in a Spinal Cord Injury Rat Model

**DOI:** 10.1111/cns.70421

**Published:** 2025-05-06

**Authors:** Chenlu Li, Zhongyue Wu, Fuxiang Chen, Chaoxian Dai, Xinyi Yang, Shumin Ye, Meng Shi, Peng Chen, Xueyan Liu, Fang Liu

**Affiliations:** ^1^ Department of Hyperbaric Oxygen National Regional Medical Center, Binhai Campus of the First Affiliated Hospital, Fujian Medical University Fuzhou China; ^2^ Fujian Provincial Key Laboratory of Brain Aging and Neurodegenerative Diseases The School of Basic Medical Sciences, Fujian Medical University Fuzhou Fujian China; ^3^ Department of Radiology First Affiliated Hospital of Fujian Medical University Fuzhou Fujian Province China; ^4^ Department of Neurosurgery Neurosurgery Research Institute, The First Affiliated Hospital, Fujian Medical University Fuzhou Fujian China; ^5^ Department of Medicinal Chemistry School of Pharmacy, Fujian Medical University Fuzhou Fujian China; ^6^ Department of Sports Medicine National Regional Medical Center, Binhai Campus of the First Affiliated Hospital of Fujian Medical University Fujian China

**Keywords:** cognitive impairment, depressive behavior, ferroptosis, GPX4, hyperbaric oxygen, proteomics, spinal cord injury

## Abstract

**Aims:**

Neuroinflammation, microglial activation, and oxidative stress contribute to neuropsychiatric deficits following spinal cord injury (SCI). Hyperbaric oxygen (HBO) therapy has demonstrated anti‐inflammatory, antioxidant, and neuroprotective properties. This study aimed to investigate the therapeutic effects and underlying mechanisms of HBO on depressive‐like behavior, cognitive impairment, and hippocampal pathology in a rat model of SCI.

**Methods:**

We employed a battery of behavioral assessments, unbiased stereological analysis, immunofluorescence staining, and biochemical assays to evaluate neuroinflammation, oxidative stress, mitochondrial damage, and iron accumulation in the hippocampus. Untargeted proteomic analysis was conducted to identify potential molecular targets of HBO. Western blotting was used to assess the activation of the Nrf2/GPX4 signaling pathway. ML385, a selective Nrf2 inhibitor, was intrathecally administered 30 min prior to daily HBO treatment to validate pathway involvement.

**Results:**

HBO treatment significantly alleviated depressive‐like behavior and cognitive deficits in SCI rats. It also suppressed M1‐type microglial activation and reduced hippocampal neuroinflammation. Additionally, HBO mitigated neuronal ferroptosis induced by SCI through activation of the Nrf2/GPX4 signaling pathway. The protective effects of HBO were abolished by coadministration of ML385, confirming the critical role of Nrf2 signaling in mediating its anti‐ferroptosis effects.

**Conclusion:**

These findings highlight ferroptosis as a key pathological mechanism in SCI‐induced hippocampal damage and suggest that HBO therapy alleviates depressive‐like behavior and cognitive impairment by targeting the Nrf2/GPX4 pathway. This study provides new insights into the therapeutic potential of HBO in managing SCI‐related neuropsychiatric dysfunction.

## Graphical abstract

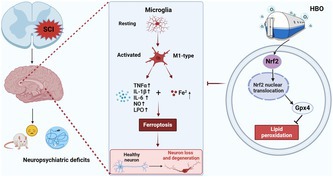

## 1 Introduction

Spinal cord injury (SCI) can have cascading effects on the central nervous system (CNS), leading to neuropsychiatric symptoms in up to 60% of SCI patients [1, 2]. These symptoms can hinder patients' physical recovery and increase mortality [3], which has notable socioeconomic implications. However, the clinical options available for treating neuropsychiatric disorders resulting from SCI are limited. Common remedies include psychotherapy, cognitive training, dietary changes, and antidepressants [4, 5, 6]. The efficacy of these approaches remains rather inconclusive [7]. This underscores the urgent need for novel therapeutic strategies with greater efficacy and mechanistic clarity.

Preclinical studies indicate SCI‐induced posttraumatic inflammation causes neuroinflammation and activates neurotoxic microglia, leading to neuronal stress, death, and neurodegeneration in specific brain regions, promoting cognitive impairment and depression [8, 9, 10, 11]. Furthermore, neuroinflammation has been shown to disrupt iron homeostasis by enhancing iron uptake and limiting iron export in neurons, which in turn exacerbates oxidative stress and promotes ferroptosis [12, 13].

Ferroptosis, a nonapoptotic cell death pathway triggered by iron‐dependent lipid peroxidation, has been reported to be associated with various neurodegenerative disorders, including Alzheimer's disease (AD) [14, 15]. While SCI‐induced neuronal ferroptosis has been reported in the sensory and motor cortex [16, 17], its presence in brain regions specifically associated with depressive‐like behavior, such as the hippocampus, remains largely unexplored.

Nuclear factor erythroid 2‐related factor 2 (Nrf2) is a pivotal transcription factor that maintains redox homeostasis and regulates cellular defense against oxidative damage. It also plays a central role in inhibiting ferroptosis by modulating the synthesis and activity of glutathione peroxidase 4 (GPX4), a key antioxidant enzyme [18]. Recent studies suggest that activation of the Nrf2/GPX4 axis exerts antidepressant‐like effects by suppressing ferroptosis [19, 20]. Therefore, pharmacological activation of this pathway may represent a promising therapeutic strategy for addressing both neuroinflammation and ferroptosis in SCI‐induced neuropsychiatric disorders.

Hyperbaric oxygen (HBO) therapy is a noninvasive and cost‐effective intervention that has shown potential in treating various neurological conditions, including traumatic brain injury, Alzheimer's disease, and vascular dementia [21, 22, 23]. HBO exerts its therapeutic effects through multiple mechanisms, such as reducing inflammation, inhibiting glial activation, improving mitochondrial function, lowering oxidative stress, and enhancing neurotrophic factor expression in the CNS. Although HBO has been shown to promote motor‐sensory recovery following SCI [24, 25], its impact on SCI‐induced neuropsychiatric symptoms has not been fully elucidated. In the present study, we established a rat model of SCI and applied a long‐term, repetitive HBO treatment regimen. Our findings indicate that HBO significantly alleviates depressive‐like behavior, cognitive deficits, and hippocampal neuroinflammation, primarily through the suppression of M1‐type microglial activation. Furthermore, proteomic analysis revealed that the therapeutic effects of HBO may be linked to the modulation of ferroptosis‐related pathways.

Building on prior research, HBO has been shown to exert antioxidant and anti‐inflammatory effects via the Nrf2 signaling pathway in pathological conditions such as middle cerebral artery occlusion, hypoxia‐ischemia brain damage, and spinal cord ischemia/reperfusion injury [26, 27, 28]. Notably, a recent in vitro study by Chen et al. demonstrated that HBO suppressed neuronal ferroptosis through the Nrf2/System Xc^−^/GPX4 axis [29]. Based on these insights, our primary objective was to investigate whether HBO exerts neuroprotective effects in SCI via modulation of the Nrf2/GPX4 signaling pathway. Ultimately, we demonstrated that HBO alleviates oxidative damage and inhibits ferroptosis, thereby mitigating SCI‐induced neuropsychiatric deficits.

## 2 Materials and Methods

### 2.1 Animals

The Experimental Animal Center of Fujian Medical University provided Sprague–Dawley adult female rats (8 weeks of age, weighed 230–250 g). We selected female rats because they exhibit higher susceptibility to major depressive disorder and cognitive impairment after central nervous system trauma [30, 31, 32]. This approach enables sex‐specific behavioral analysis and addresses the underrepresentation of females in biomedical research [33], thereby advancing personalized medicine.

Animals were housed under a 12 h light–dark cycle at 23°C ± 2°C, 53% ± 2% relative humidity, in separate cages with free access to rodent chow and water. The Laboratory Animal Ethics Committee of the First Affiliated Hospital of Fujian Medical University approved all animal experiments (IACUC FJMU 2022–0879, Fuzhou, China), which were performed according to the National Institutes of Health guidelines.

### 2.2 Experimental Design

We performed two experiments.

#### 2.2.1 First Experiment

After 1 week of acclimatization, 96 rats were randomly assigned to the following six groups (*n* = 16 per group): Control (Ctrl); Sham‐operated (Sham); Sham‐operated with sham HBO treatment (Sham + sham HBO); Spinal cord injury model (SCI); Spinal cord injury model + sham HBO treatment (SCI + sham HBO) and spinal cord injury model followed by 40‐day HBO treatment (HBO).

**FIGURE 1:**
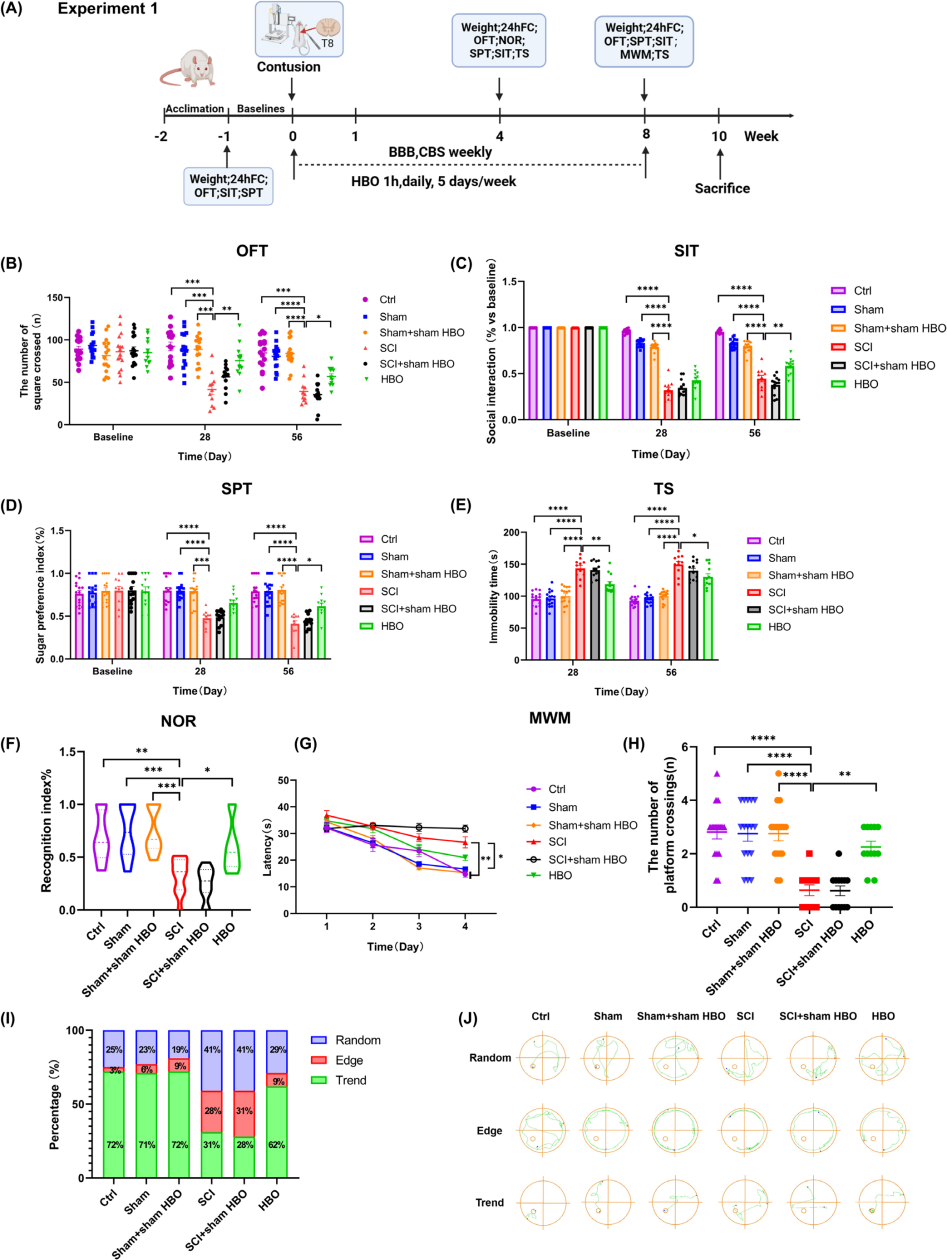
Effects of HBO on the prevention of depressive behavior and cognitive impairment of SCI rats. (A) Timeline of Experiment 1. 24hFC = 24 h food consumption, BBB = Basso, Beattie and Bresnahan test; CBS = Combined behavioral score; OFT = Open field test; SIT = Social interaction test; SPT = Sugar preference test; NOR = Novel object recognition; MWM = Morris water maze; TS = Tail suspension. (B) The number of squares crossed in OFT. (C) The social interaction test. (D) The sucrose preference test. (E) The immobility time in TS. (F) Recognition index in NOR. (G) The latency to finding the hidden platform during the training days in the MWM test. (H) The number of hidden platform crossings for the probe test. (I) The percent of search strategies taken by each group. (J) Representative MWM trajectory of search strategies for all groups. *p < 0.05, **p < 0.01, ***p < 0.001, ****p < 0.0001, versus SCI group. Data are presented as means ± SEM, *n* = 11–16; Significance was determined by mixed‐effect model analysis with post hoc Tukey tests or two‐way repeated measures ANOVA followed by Bonferroni post hoc test or one‐way ANOVA with post hoc Tukey tests.

Baseline behavioral assessments were conducted before surgery. A total of 48 rats underwent SCI contusion; 12 rats were excluded due to death postinjury (*n* = 9) or failure of SCI induction (*n* = 3). The experimental timeline is shown in Figure 1A.

#### 2.2.2 Second Experiment

Rats (*n* = 8 per group) were randomly assigned to: Sham; SCI; HBO; and HBO + ML385 (10 μg/d, intrathecally for 11 consecutive days). ML385 was administered in the SCI groups 30 min before HBO treatment, as shown in Figure 6A.

### 2.3 SCI Model

Acute SCI surgeries were performed using the modified Allen's method, as previously described [34]. We used the New York University Impactor III to smash the exposed cord (10 g × 50 mm) to cause a severe contusion. After contusion, the spinal cord became hyperemic and edematous, generating a 5 mm‐diameter lesions. All rats were given daily gentamicin intramuscular injections and regular bladder massages twice a day to help them urinate until spontaneous voiding returned.

### 2.4 HBO Administration

After SCI operation, a randomized block experimental design was used to allocate rats to the HBO group. Rats in the HBO group received 60 min of HBO (PO_2_ = 95%–100% at 2.0 atm absolute; ATA) in an animal HBO chamber (DC0325J‐X, Yantai Moon Group Co. Ltd., Shandong, China). The procedure was started 2 h after SCI, 60 min per session, daily 5 days/week, until day 56 (total 40 sessions). The therapy protocol was conducted as described in our previous study [34]. Rats in Ctrl, Sham, and SCI groups were in ambient air outside the chamber. Those in Sham + sham HBO and SCI + sham HBO groups had “sham HBO”: 60‐min sessions for 40 times in a chamber without actual HBO, just mimicking conditions as control for nonspecific chamber effects [35].

### 2.5 Psychological Well‐Being Evaluation

#### 2.5.1 Open Field Test

The open field test (OFT) was performed as described [36] to assess anxiety‐like behavior. Baseline activity levels were collected 5 days prior to surgery, and behavior was scored using the Video Analysis System (Jiliang Software, JLBehv‐LAM‐1, Shanghai, China). The number of squares crossed in a 5‐min test session was recorded as a measure of psychomotor activity.

#### 2.5.2 Social Interaction Test

To assess social behavior, we measured social exploratory behavior as described [37]. The length of time that the experimental rats spent socially observing the juvenile (such as anogenital sniffing and trailing) was recorded. The results were compared to the baseline measurements taken before the spinal injury, which were considered as 100% of the results.

#### 2.5.3 Sucrose Preference Test

Sucrose preference test (SPT) was tested as described with some modifications [38] (detailed information shown in Supporting Information). Decreased sugar preference is a manifestation of anhedonia indicating depression‐like behavior.

Weight gain and food consumption were also collected at baseline and at the 4th and 8th week before SPT, as an index of appetite deviation, which is associated with depression in the clinical population. Thirty grams of food was given at 17:00, food weight was recorded at 17:00 the next day, animals were weighed at the same time, and 24 h food consumption = [(total food − food surplus)/ body weight × 100%].

#### 2.5.4 Tail Suspension Test

The tail suspension test (TS) was used to evaluate symptoms of depression. Rats were taped to a horizontal bar at a height of 50 cm and suspended by their tails. The visual track software recorded behavior for 6 min and calculated the amount of time spent immobile.

### 2.6 Cognitive Function Evaluation

#### 2.6.1 Novel Object Recognition

The novel object recognition (NOR) test, which is less dependent on locomotion, was conducted on week 4 after contusion, as described [39] (detailed information shown in Supporting Information).

#### 2.6.2 Morris Water Maze

Spatial learning and memory performance were assessed using the standard Morris water maze (MWM) tests. The protocols were similar as described [40].

To diminish the impact of injured locomotion, we performed the maze test at the 10th week after SCI, when neither the SCI nor the HBO group showed significantly worse locomotor activity (swimming speed shown in Figure S1H) compared with the Ctrl and Sham rats. Thus, these results validated cognitive evaluation. In addition, to better eliminate the influence of individual motor ability differences, we carefully analyzed the swimming track map of each animal and used the following three search strategies to rigorously evaluate cognitive level (detailed information shown in Supporting Information). On the fourth training day of the maze test, the search strategies were examined in each of four trials. The percent of each strategy in each group was calculated.

### 2.7 Histopathology

Six rats per group were sacrificed under deep pentobarbital anesthesia (100 mg/kg, Ceva, i.p.) and perfused transcranially with 300 mL of 0.1 M ice‐cold phosphate buffer (pH 7.4) followed by the same volume of 4% phosphate‐buffered paraformaldehyde (pH 7.4). The rat brains were carefully removed.

#### 2.7.1 Nissl Staining

Brain tissues were fixed in 4% paraformaldehyde, dehydrated, embedded in paraffin, soaked in 0.5% toluidine blue, stained for 5 min, washed with water, and differentiated with 1% glacial acetic acid. Images were observed by microscopy and analyzed by CaseView software. Two mutually uncrossed view fields were arbitrarily intercepted in the hippocampal region at a magnification of 20×. The number of cells containing normal Nissl bodies in the intercepted images was analyzed by Image J software [41].

#### 2.7.2 Perl's Prussian Blue Staining

The brain tissue sections were stained with Prussian Blue Iron Stain Kit (GP1068, Service bio, Wuhan, China) to measure iron content, according to the manufacturer's instructions. Images were obtained with a light microscope (E100, Nikon, Japan).

#### 2.7.3 Transmission Electron Microscope

Rats were anesthetized and perfused with 2% paraformaldehyde, 2% glutaraldehyde in 0.1 M sodium cacodylate. Hippocampus samples were collected from removed brains (1 mm^3^) and cut into sections (70–90 nm). After being postfixed in 2% osmium tetroxide, brain samples were stained with 2% uranyl acetate, dehydrated in ethanol, and then embedded in Eponate. Finally, samples were placed on copper slot grids, followed by being stained with 2% uranyl acetate and lead citrate. The mitochondrial morphology was visualized using a transmission electron microscope (7700; Hitachi, Japan).

#### 2.7.4 Immunohistochemistry

Paraffin‐embedded tissue sections were deparaffinized with xylene and rehydrated through a graded ethanol series. Antigen retrieval was performed in Tris‐EDTA buffer (ServiceBio) with heat. Endogenous peroxidase activity was blocked with 3% hydrogen peroxide for 25 min at 37°C, followed by blocking with 3% BSA for 30 min. Sections were incubated with iNOS primary antibodies (1:500, GB11119‐100, ServiceBio) overnight at 4°C, then with HRP‐conjugated secondary antibodies (1:200, ServiceBio) for 50 min at room temperature. Visualization was achieved using a DAB kit and counterstaining with hematoxylin. Positive cells were quantified using ImageJ at 400× magnification.

#### 2.7.5 Immunofluorescence

Brains were fixed in 4% PFA at 4°C for 24 h. Specimens for paraffin sections were dehydrated through graded ethanol (70%, 80%, 95%, 100%), infiltrated with xylene, and embedded in paraffin. For cryostat sections, specimens were dehydrated in 30% sucrose solution for 48 h until sinking and sectioned at 40 μm using a cryostat microtome. For immunofluorescent staining, cryostat and paraffin sections were permeabilized and blocked in PBST (PBS + 0.1% Triton X‐100) with 5% normal serum for 1 h at room temperature. The primary antibodies: anti‐NeuN (1:200, E3M5P, CST); anti‐Nrf2 (1:200, EP1808Y, Abcam); anti‐GPX4 (1:200, ab125066, Abcam); anti‐Iba1 (1:1000, 019–19,741, Wako); anti‐CD86 (1:200, BM4121, Boster); anti‐PSD95 (1:200, GB11277‐100, ServiceBio); anti‐synaptophysin (1:500, GB15814‐100, ServiceBio) were incubated at 4°C overnight. After washing, samples were treated with secondary antibodies for 2 h at room temperature, followed by DAPI staining (2 μM) for 10 min. Paraffin sections underwent dewaxing, rehydration, and antigen retrieval before staining.

Imaging was performed using a fluorescence microscope (Leica Microsystems, Germany) for cryostat sections and a Nikon DS‐U3 system for paraffin sections. Quantification was conducted with Image J software.

### 2.8 Protein Sample Preparation for Proteomic Analysis

Specimens were collected at the 10th week after SCI contusion. The both sides of hippocampus tissues of four rats per group were isolated from the brain on ice and were rapidly snap‐frozen in liquid nitrogen and stored at −80°C until analysis.

The detailed procedures and information of DIA Quantitative Proteomic Analysis and bioinformatics analysis were described in Supporting Information Appendix 1.

### 2.9 Biochemical Measurements

#### 2.9.1 Assessment of Markers for Oxidative Stress and Antioxidant Capacity

The enzymatic activity of superoxide dismutase (SOD), glutathione (GSH), and glutathione peroxidase (GSH‐Px), and the content of lipid peroxide (LPO) and nitric oxide (NO) were measured with commercial kits (Jiancheng and Beyotime, China). The absorbances of the colored substances were measured with a spectrophotometer.

#### 2.9.2 Enzyme‐Linked Immunosorbent Assay (ELISA)

The levels of TNF‐α, IL‐1β, and IL‐6 in serum and brain tissues were measured with ELISA kits (ThermoFisher Scientific, China), and the concentrations of ferritin heavy chain (FTH) and Fe^2+^ were measured with ELISA kits (Jiancheng, Nanjin, China; E‐BC‐K881‐M, Elabscience, China). The levels of TLR4 and NF‐κB were measured by ELISA kits (Ruixin, China). Experimental procedures were executed according to the manufacturer's specifications and repeated three times. The absorbance of the samples was measured at 450 nm using a 96‐well microplate reader (Varioskan MultiSkan FC, ThermoFisher Scientific, USA).

### 2.10 Immunoblotting

Total protein was extracted from rat hippocampus using the RIPA Kit (r0010, Solarbio, China). Protein concentration was measured by the BCA method. Quantitative analysis was performed in accordance with the different concentrations. Protein was resolved by 10% SDS‐PAGE and transferred by electrophoresis to a nitrocellulose membrane. The membrane was blocked by incubation with 5% skim milk for 2 h at ambient temperature and then incubated at 4°C with primary antibodies: anti‐NeuN (rabbit, 1:3000, GB11138, Servicebio, China), anti‐Iba1 (rabbit, 1:1000, ab5076, Abcam, UK), anti‐CD86 (rabbit, 1:5000, ET1606‐50, HUABIO, China), anti‐iNOS (rabbit, 1:2000, 18,985‐1‐AP, Proteintech, China), anti‐PSD95 (rabbit 1:1000, CST, USA), anti‐synaptophysin(rabbit, 1:5000, ab32127, Abcam, UK), anti‐Nrf2 (rabbit, 1:3000, ab62352, Abcam, UK), anti‐HO‐1 (rabbit, 1:1000, 10,701‐1‐AP, Proteintech, China), anti‐GPX4(rabbit, 1:1000, ab125066, Abcam, Cambridge, UK), anti‐BDNF(rabbit, 1: 2000, GB11559, Servicebio, China), anti‐TrkB (rabbit, 1:2000, GB11295‐1, Servicebio, China), anti‐p‐CREB(rabbit, 1: 3000, GB114684, Servicebio, China), anti‐CREB(rabbit, 1: 3000, GB111052, Servicebio, China), anti‐HIF‐1α (rabbit, 1:1000, AB179483, Abcam, UK), and anti‐β‐actin (mouse, 1:2000, 8H10D10, CST, USA or GB15001, Servicebio, China), and anti‐lamin B1 (mouse, 1:2000, ab16048, Abcam, UK). The membranes were then incubated with appropriate secondary antibodies. The immunoreactivity was detected by enhanced chemiluminescence and visualized using Image Lab software (Bio‐Rad, USA). The intensities of the protein bands were quantified by Image J software (National Institutes of Health, USA) and the relative expression levels of proteins were normalized by the ratio of the target protein to β‐actin.

### 2.11 Statistical Analysis

Data analysis was performed using GraphPad Prism 8.0 software (GraphPad, La Jolla, CA, USA). The Shapiro–Wilk test was employed to evaluate the normality of the data. Data were presented as means ± SEM. Weight and 24‐h food consumption data were analyzed using a mixed‐effect model followed by Dunnett's T3 post hoc test when the number of samples per group was different. The water maze escape latency was analyzed with two‐way repeated measures ANOVA followed by Bonferroni post hoc test. The differences between groups were compared using one‐way ANOVA followed by Tuckey's post hoc tests for normally distributed data and Kruskal–Wallis test for non‐normally distributed data. All values of *p* < 0.05 were defined as statistically significant.

## 3 Results and Discussion

### 3.1 HBO Prevented Depressive‐Like Behavior and Cognitive Impairment Following Spinal Cord Injury

To evaluate whether early and sustained HBO treatment can attenuate neuropsychiatric deficits following chronic SCI, we conducted a battery of depressive and cognitive‐behavioral tests.

As shown in Figure 1, no significant differences were observed among the Ctrl, Sham, Sham + sham HBO, SCI, SCI + sham HBO, and HBO groups in all behavioral tests conducted prior to SCI induction. However, starting from the fourth‐week postinjury, SCI rats without HBO treatment displayed pronounced depressive‐like behaviors. Specifically, they showed significantly reduced locomotor activity in the open field test (OFT) (Figure 1B), decreased food intake and body weight gain (Figure S1A,B), reduced social interaction (Figure 1C), lower sucrose preference (Figure 1D), and increased immobility time in the tail suspension test (TST) (Figure 1E). These observations are consistent with previous reports indicating the early emergence of depressive‐like behaviors in female SCI models [42]. Notably, HBO treatment effectively mitigated these behavioral deficits.

To assess cognitive performance, we employed the novel object recognition (NOR) and Morris water maze (MWM) tests. At 4 weeks post‐SCI, all groups exhibited no preference during the NOR sample phase (Figure S1C). However, in the test phase, the SCI and SCI + sham HBO groups showed significantly decreased exploration of the novel object (Figure S1D) and a reduced recognition index (Figure 1F), indicating short‐term memory impairment. HBO treatment significantly reversed these deficits.

At 8 weeks postinjury, MWM testing was performed to evaluate spatial learning and memory (Figure 1G–J). During the training phase, SCI rats without HBO treatment required longer times to locate the hidden platform, whereas rats in the HBO group showed a reduction in escape latency, although this difference was not statistically significant (Figure 1G). In the probe trial, rats in the HBO group spent more time in the target quadrant (Figure S1G) and crossed the platform more frequently (Figure 1H) compared to untreated SCI rats, who demonstrated significantly impaired spatial memory.

Moreover, search strategy analysis revealed that SCI rats predominantly relied on edge‐swimming strategies, indicative of spatial learning deficits. In contrast, HBO‐treated rats exhibited a substantial shift toward trend (goal‐directed) strategies, with 60% using trend strategies and only 9% relying on edge patterns (Figure 1J), suggesting improved cognitive flexibility. Consistently, the HBO group also showed a shorter total swimming distance than the SCI and SCI + sham HBO groups (Figure S1I), further supporting enhanced navigational efficiency through strategy optimization.

Collectively, these findings demonstrate that SCI impairs both spatial and nonspatial memory, while HBO therapy effectively attenuates depressive‐like behaviors and cognitive impairments in this chronic SCI model.

### 3.2 HBO Mitigated SCI‐Induced Hippocampal Neuroinflammation by Inhibiting M1‐Type Microglia Activation and Promoted Neuronal and Synaptic Recovery

As previously reported [8, 10], SCI triggers spinal‐brain inflammatory signaling that activates microglia. Among these, neurotoxic M1‐type microglia release proinflammatory mediators that exacerbate neuronal damage [43, 44]. Therefore, attenuating M1‐type microglial activation is essential to prevent hippocampal neuroinflammation following SCI.

**FIGURE 2:**
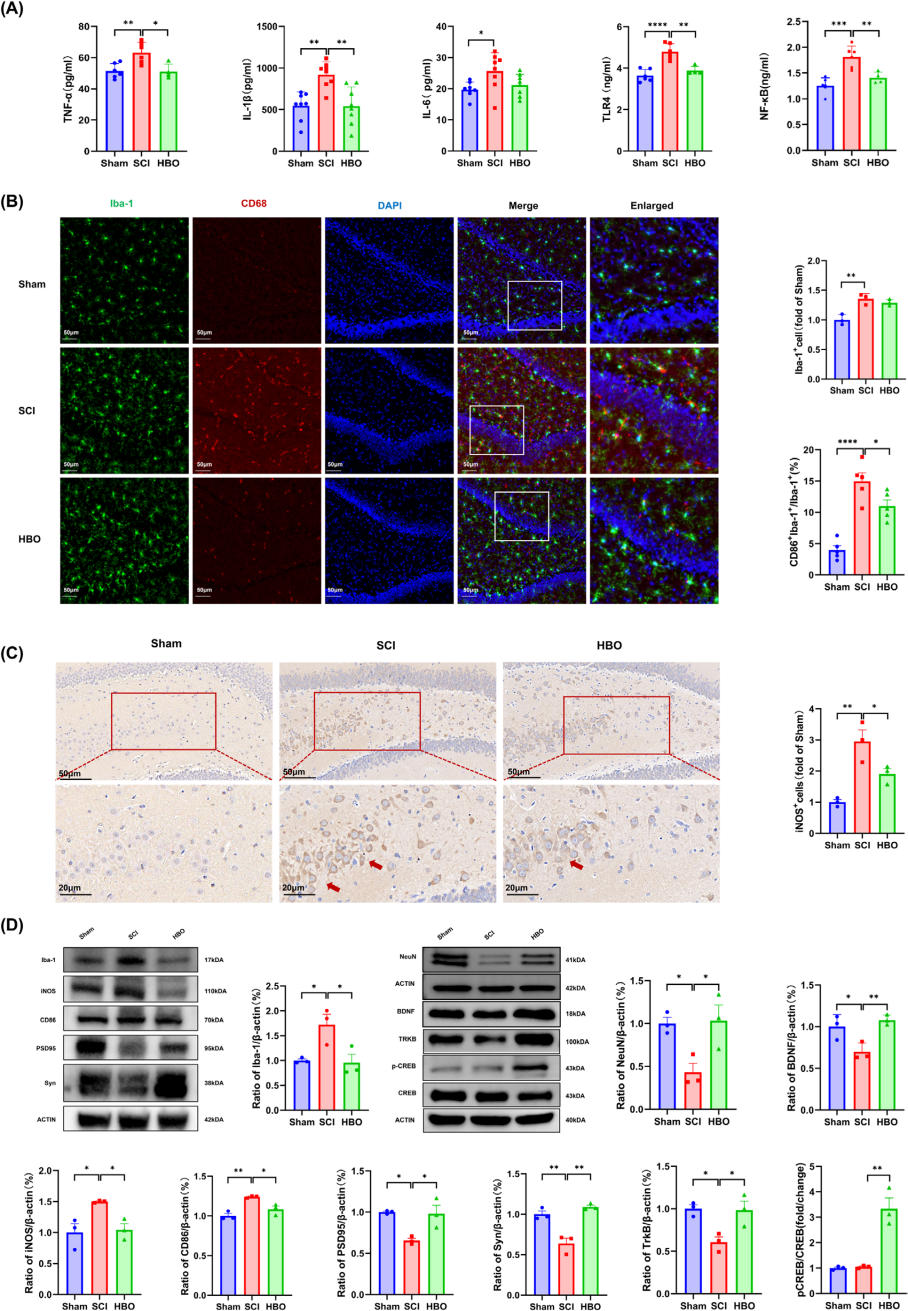
HBO prevented SCI‐induced neuroinflammation by inhibiting activation of M1‐type microglia and promoting neuronal and synaptic recovery. (A) Quantification of hippocampal TNF‐α, IL‐1β, IL‐6, TLR4, and NF‐κB levels by ELISA at 10 weeks post‐SCI. (B) Representative double immunofluorescence images and quantitative analysis of Iba‐1‐positive microglia and Iba‐1/CD86 double‐positive M1‐type microglia in the hippocampus. Scale bars = 50 μm. (C) Representative immunohistochemical images and quantification of iNOS‐positive cells in the hippocampus. Scale bars = 50 μm; magnified inset scale bar = 20 μm. (D) Representative western blot bands and quantification of hippocampal protein expression levels of Iba‐1, CD86, iNOS, PSD95, Syn, and components of the BDNF/TrkB/CREB signaling pathway. All data were normalized to the expression levels of the sham group. Values are presented as mean ± SEM (*n* = 3–6 per group). *p < 0.05, **p < 0.01, ***p < 0.001, ****p < 0.0001 versus SCI group. Statistical analysis was performed using one‐way ANOVA followed by Tukey's post hoc test.

Consistent with previous studies [9, 40, 42], we observed significantly elevated levels of proinflammatory cytokines TNF‐α, IL‐1β, and IL‐6 in the hippocampus of SCI rats compared to Sham controls (Figure 2A). It is well established that cytokine overproduction activates microglia and contributes to neuronal injury and synaptic dysfunction, ultimately leading to depressive‐like behavior [45]. Remarkably, 40 sessions of HBO treatment significantly reduced hippocampal levels of TNF‐α and IL‐1β, and suppressed activation of the TLR4/NF‐κB signaling pathway; however, IL‐6 levels remained unchanged.

To further assess M1‐type microglial activation, we performed double‐immunofluorescence staining for Iba‐1 (a general microglial marker) and CD86 (an M1‐specific marker), as well as immunohistochemical staining for inducible nitric oxide synthase (iNOS). SCI rats displayed a substantial increase in CD86/Iba‐1 double‐positive microglia in the hippocampus compared to Sham and HBO‐treated groups (Figure 2B). Similarly, the number of iNOS‐positive cells was markedly elevated in the SCI group (Figure 2C), indicating increased M1‐type activation and neuroinflammation. HBO treatment significantly reduced the expression of both markers, suggesting effective inhibition of M1 microglial polarization. These findings were corroborated by immunoblotting results, which showed upregulation of CD86 and iNOS in SCI rats, both of which were significantly suppressed by HBO (Figure 2D).

We next employed Nissl staining to assess neuronal integrity. Compared with Sham rats, SCI animals exhibited disrupted neuronal morphology in the hippocampus, characterized by sparse neuronal distribution, irregular arrangement, and reduced or absent Nissl bodies, which indicated neuronal degeneration (Figure S2A). HBO treatment alleviated these histological abnormalities, restoring neuronal density and Nissl substance, which underscores its neuroprotective effect. Neuronal damage and synaptic dysfunction are closely linked, forming a pathological cycle that is particularly pronounced in depression and Alzheimer's disease [46].

Brain‐derived neurotrophic factor (BDNF) and its receptor TrkB play pivotal roles in synaptic plasticity. Their interaction activates downstream pathways that phosphorylate CREB, promoting transcription of genes essential for dendritic spine formation and long‐term potentiation: critical processes for learning, memory, and neural circuit repair [46, 47]. Additionally, synaptic proteins such as PSD95 and synaptophysin (Syn) are indispensable for maintaining synaptic function, and their dysregulation is implicated in both cognitive impairment and depression [48].

To assess HBO's impact on synaptic integrity and plasticity, we performed double‐immunofluorescence staining (Figure S2B,C) and immunoblotting (Figure 2D) for BDNF, NeuN, PSD95, and Syn. HBO treatment significantly increased both fluorescence intensity and protein expression of these markers, all of which were reduced in SCI rats, indicating enhanced neuronal health and synaptic recovery. Moreover, HBO upregulated the BDNF/TrkB/CREB signaling axis, suggesting improved synaptic strength and long‐term potentiation.

In summary, HBO exerted potent anti‐inflammatory effects, restored hippocampal neuronal structure, and improved synaptic plasticity in SCI rats. However, the precise molecular mechanism by which HBO alleviates SCI‐induced neuropsychiatric deficits remains to be fully elucidated. Therefore, we employed untargeted proteomic analysis to identify potential signaling pathways involved in HBO‐mediated recovery.

### 3.3 HBO Modulated Hippocampal Protein Expression Profiles in SCI Rats

**FIGURE 3:**
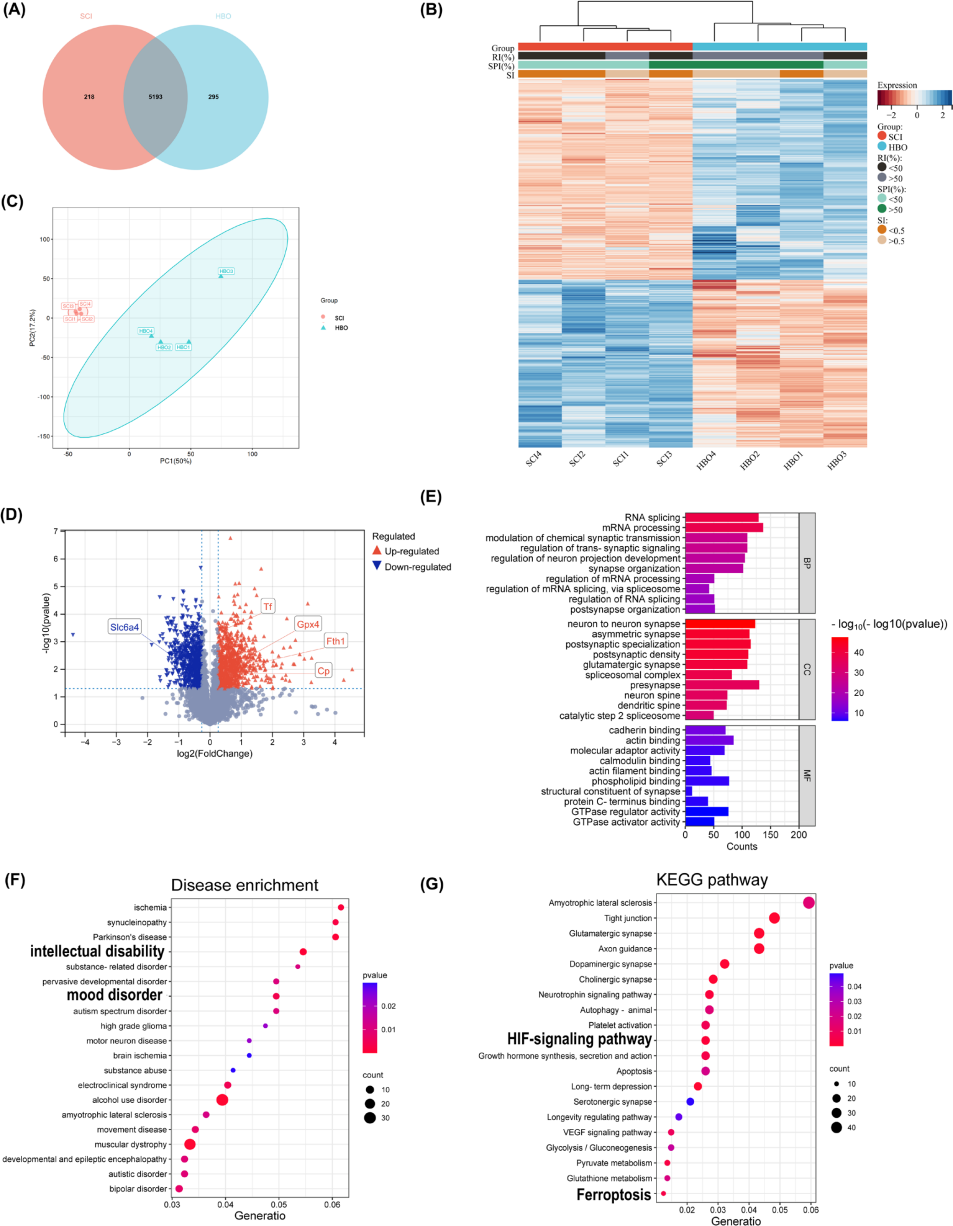
Differential protein expression and bioinformatic analysis in the hippocampus of HBO‐treated SCI rats with depressive‐like behavior. (A) Venn diagram showing the overlap of differentially expressed proteins between the SCI and HBO groups (*n* = 4 per group). (B) Principal component analysis (PCA) demonstrating group separation based on overall proteomic profiles. (C) Hierarchical clustering of proteins with significant differential expression (*p* < 0.05, |Log_2_ fold change| ≥ 0.263) between groups. (D) Volcano plot displaying significantly upregulated (red) and downregulated (blue) proteins in the HBO group versus the SCI group. (E) Gene ontology (GO) enrichment analysis categorized into biological process (BP), cellular component (CC), and molecular function (MF). (F) Kyoto Encyclopedia of Genes and Genomes (KEGG) pathway enrichment analysis highlighting major signaling pathways. (G) Disease ontology (DO) enrichment analysis showing associations with neuropsychiatric and neurodegenerative conditions.

At 10 weeks post‐SCI, hippocampal tissues were collected for proteomic analysis using LC–MS/MS. From eight biological replicates, we identified 5706 nonredundant proteins, of which 5193 were shared between the SCI and HBO groups (Figure 3A). To ensure analytical rigor, only the 4930 proteins detected in at least three samples per group were retained for differential expression analysis.

Hierarchical clustering revealed distinct protein expression profiles between the SCI and HBO groups, suggesting treatment‐related differences (Figure 3B). Similarly, principal component analysis (PCA) demonstrated clear separation between groups, further supporting the impact of HBO on hippocampal protein expression (Figure 3C).

A total of 1633 differentially expressed proteins (DEPs) were identified using a threshold of *p* < 0.05 and |Log2 fold change| ≥ 0.263. Of these, 892 proteins were upregulated and 741 were downregulated in the HBO group compared to the SCI group (Figure 3D), indicating that HBO elicited broad proteomic alterations in the hippocampus.

To explore the biological significance of these DEPs, we performed gene ontology (GO) enrichment analysis across three categories: Biological process (BP), cellular component (CC), and molecular function (MF). In the BP category, DEPs were primarily involved in the regulation of neuron projection development, trans‐synaptic signaling, and modulation of chemical synaptic transmission. CC terms included neuron‐to‐neuron synapses, asymmetric synapses, glutamatergic synapses, and postsynaptic specializations. For MF, enriched terms included cadherin binding, actin filament binding, calmodulin binding, and molecular adaptor activity (Figure 3E).

To further investigate potential disease associations, we performed Disease Ontology (DO) enrichment analysis. DEPs were significantly associated with mood disorders, autism spectrum disorders, motor neuron disease, intellectual disability, Parkinson's disease, amyotrophic lateral sclerosis (ALS), muscular dystrophy, and alcohol use disorders (Figure 3F).

KEGG pathway analysis revealed significant enrichment in pathways relevant to neurological and psychiatric disorders, including ALS, glutamatergic, dopaminergic, and cholinergic synapses, neurotrophin signaling, axon guidance, autophagy, HIF‐1 signaling, glutamine metabolism, serotonergic synapses, and notably, ferroptosis (Figure 3G). Among these, the ferroptosis pathway was particularly intriguing due to the paucity of literature linking it to SCI‐induced neuropsychiatric deficits.

Further analysis of ferroptosis‐related proteins revealed that HBO treatment significantly increased the expression of GPX4 and FTH: two critical regulators of ferroptosis (Figure S3). These results suggest that HBO may exert its antidepressant and cognitive‐protective effects, at least in part, through modulation of ferroptosis and related metabolic pathways.

Taken together, the proteomic data support the hypothesis that HBO influences hippocampal function in SCI by regulating glutathione metabolism, HIF‐1 signaling, and ferroptosis. These findings provide novel mechanistic insights into how HBO may alleviate depressive‐like behavior and cognitive impairment following SCI.

### 3.4 HBO Attenuated Oxidative Stress and Mitochondrial Damage, Thereby Inhibiting Ferroptosis in the Hippocampus of SCI Rats

Ferroptosis is a regulated form of cell death initiated by iron‐dependent lipid peroxidation and oxidative imbalance [14]. To investigate whether HBO mitigates oxidative stress‐induced lipid peroxidation in SCI, we evaluated the levels of several oxidative stress markers in the hippocampus, including lipid peroxidation (LPO), nitric oxide (NO), superoxide dismutase (SOD), glutathione (GSH), and glutathione peroxidase (GSH‐Px).

**FIGURE 4:**
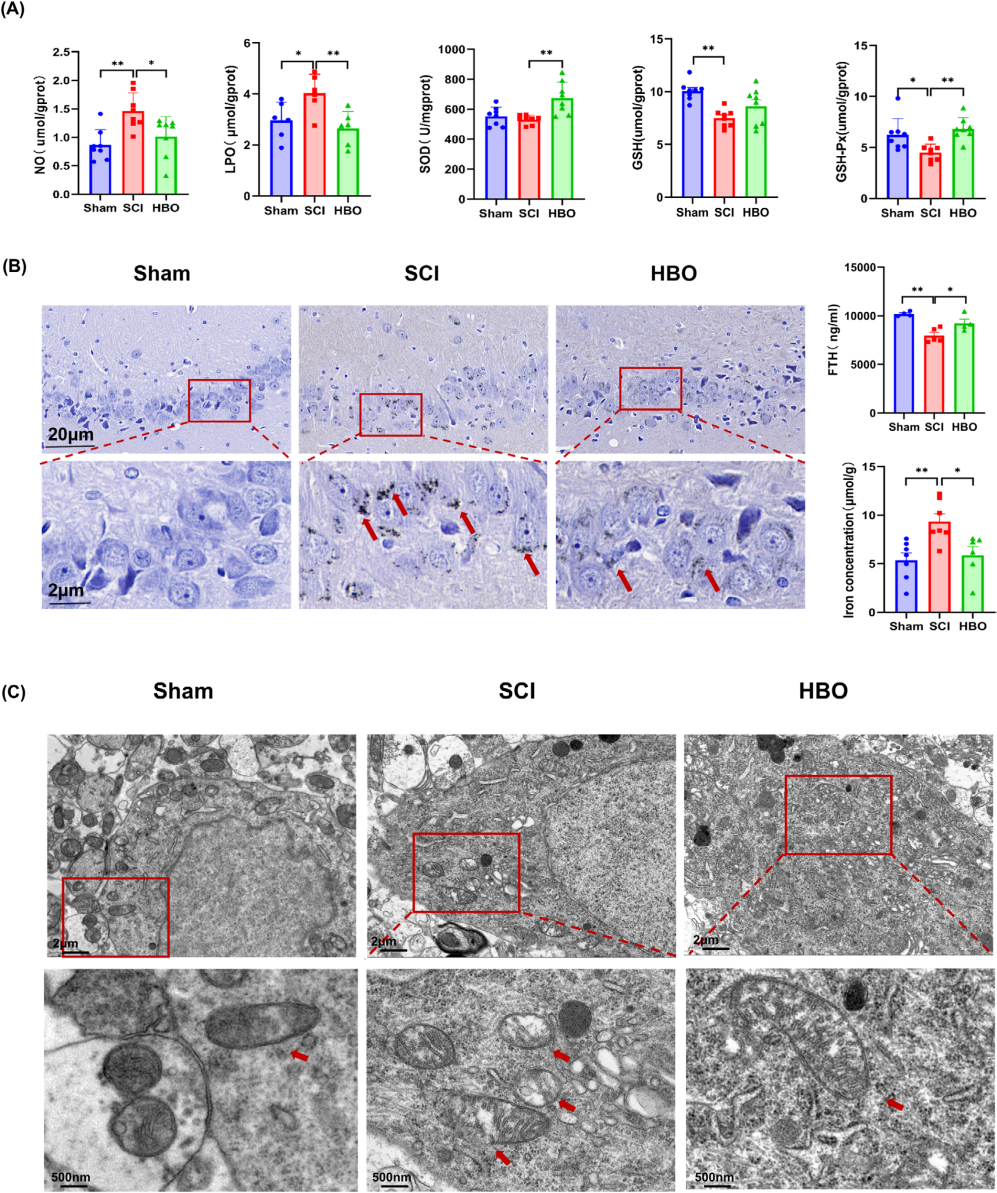
HBO inhibited iron accumulation and alleviates mitochondrial damage by mitigating the accumulation of oxidation products. (A) HBO significantly decreased SCI‐induced NO and LPO, while increasing GSH, GSH‐Px, and SOD levels in the brain at 10 weeks postcontusion. (B) Iron deposition (indicated by red arrows) in the hippocampus was assessed using Perl's Prussian blue staining. Hippocampal Fe^2+^ concentration and FTH expression were quantified by ELISA at 10 weeks post‐SCI. (C) Representative transmission electron microscopy images of hippocampal neurons showing mitochondrial ultrastructure. Scale bars: 2 μm (upper panel), 500 nm (lower panel). Data are presented as mean ± SEM (*n* = 4–8 per group). *p < 0.05, **p < 0.01, ***p < 0.001, ****p < 0.0001 versus SCI group. Statistical significance was determined by one‐way ANOVA followed by Tukey's post hoc test.

ELISA results revealed that SCI significantly elevated hippocampal levels of LPO and NO, while SOD and GSH‐Px levels were markedly reduced compared to the Sham group (Figure 4A). HBO treatment effectively reversed these changes, significantly reducing LPO and NO levels and restoring SOD and GSH‐Px activities. These findings suggest that HBO robustly suppresses lipid peroxidation and enhances endogenous antioxidant defenses. These antioxidant effects are consistent with previous studies demonstrating similar protective actions of HBO [49, 50, 51].

Iron metabolism is a critical regulator of ferroptosis. We observed increased iron accumulation and reduced levels of FTH in the hippocampus of SCI rats (Figure 4B), indicating iron overload and impaired storage. HBO treatment significantly reversed both effects: decreasing iron accumulation and restoring FTH levels, thereby promoting iron sequestration in a soluble, nontoxic form and reducing the pool of free, redox‐active iron.

To further assess ferroptotic pathology, we performed transmission electron microscopy to examine mitochondrial ultrastructure in hippocampal neurons. SCI rats exhibited characteristic mitochondrial abnormalities, including reduced mitochondrial numbers, membrane collapse, volume shrinkage, and increased vacuolation: hallmarks of ferroptosis‐associated mitochondrial damage (Figure 4C). In contrast, HBO treatment preserved mitochondrial number and morphology, maintaining intact membranes and regular structure.

Together, these findings indicate that HBO alleviates oxidative stress, restores redox balance, maintains mitochondrial integrity, and mitigates ferroptosis in the hippocampus following SCI.

### 3.5 HBO Suppressed Ferroptosis by Activating Nrf2/GPX4 Signaling Pathway

Nrf2, an antioxidant, regulates the synthesis of GPX4 protein and has a key antiferroptosis function [52, 53]. Although activation of Nrf2 has demonstrated significant anti‐ferroptosis effects in cancer models, research investigating its potential therapeutic role in mitigating ferroptosis within the field of neuropsychiatric disorders remains limited and underexplored [53]. Notably, long‐term HBO treatment has been reported to activate Nrf2 and its downstream targets [54, 55]. For example, Chen et al. demonstrated that HBO protects HT22 and PC12 cells from oxygen–glucose deprivation/reperfusion injury by inhibiting ferroptosis through the Nrf2/System Xc^−^/GPX4 axis [29].

Based on these findings and our proteomic data, we hypothesized that HBO may enhance GPX4 expression via Nrf2 activation and nuclear translocation, thereby attenuating ferroptosis in the hippocampus of SCI rats (Figure 3G, Figure S3). To verify this, we examined the Nrf2/GPX4 signaling pathway using immunofluorescence and western blot analysis.

**FIGURE 5:**
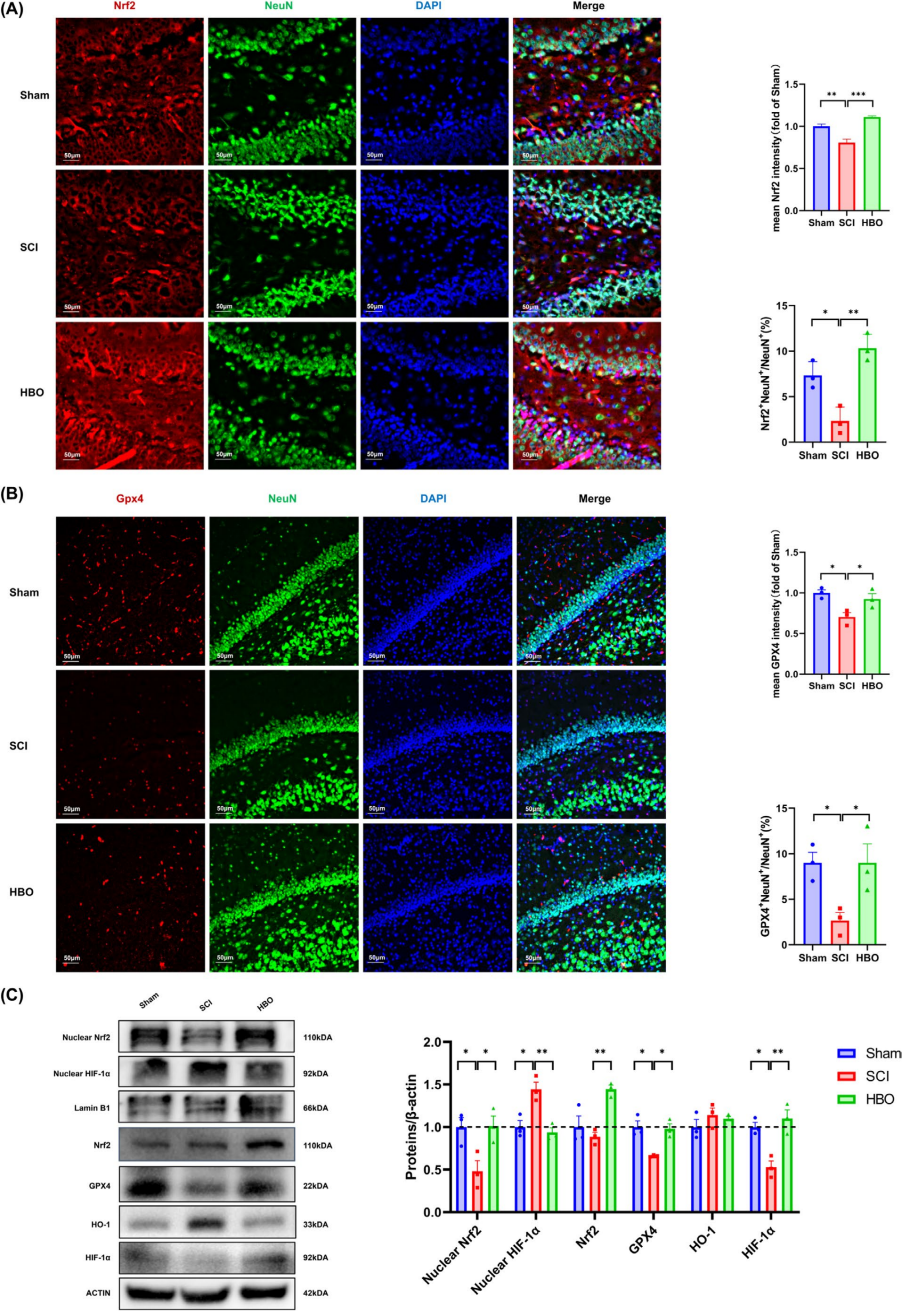
HBO suppressed ferroptosis by activating the Nrf2/GPX4 signaling pathway. (A) Representative immunofluorescence images of Nrf2/NeuN co‐labeling in the hippocampus, with corresponding quantification of Nrf2 fluorescence intensity and the number of Nrf2/NeuN double‐positive cells. Scale bar = 50 μm. (B) Representative immunofluorescence images of GPX4/NeuN co‐labeling in the hippocampus, with quantification of GPX4 fluorescence intensity and GPX4/NeuN double‐positive cell counts. Scale bar = 50 μm. (C) Representative western blot bands and quantification of hippocampal protein expression levels of nuclear Nrf2, nuclear HIF‐1α, total Nrf2, HO‐1, total HIF‐1α, and GPX4. All protein levels were normalized to those of the sham group. Data are presented as mean ± SEM (*n* = 3–8 per group). *p < 0.05, **p < 0.01, ***p < 0.001, ****p < 0.0001 versus SCI group. Statistical analysis was performed using one‐way ANOVA followed by Tukey's post hoc test.

Immunofluorescence staining revealed that SCI significantly reduced the fluorescence intensity of Nrf2 and GPX4 in the hippocampus, as well as the number of Nrf2/NeuN and GPX4/NeuN double‐positive cells. HBO treatment restored these levels to near‐normal (Figure 5A,B). Consistently, immunoblotting showed significantly decreased levels of nuclear and total Nrf2, as well as GPX4 in the SCI group, all of which were markedly upregulated following HBO treatment (Figure 5C), supporting our hypothesis that HBO activates Nrf2/GPX4 signaling to suppress ferroptosis.

Interestingly, our study found that heme oxygenase‐1 (HO‐1), a classic downstream target of Nrf2, did not show a similar expression pattern. Although HO‐1 levels were slightly elevated in the SCI group, this change did not reach statistical significance, and HBO did not significantly alter HO‐1 expression. In contrast, total HIF‐1α expression was significantly reduced in SCI rats. HIF‐1α is a key transcriptional regulator in hypoxia‐related responses and has recently been implicated in ferroptosis regulation [56, 57]. It has been reported that chronic oxidative stress promotes HIF‐1α nuclear translocation, which can bind to the promoter of *Hmox1*, thereby upregulating HO‐1 expression. Paradoxically, this may promote ferroptosis by increasing intracellular ferrous iron, enhancing lipid peroxidation and oxidative stress [58, 59].

Based on these insights, we propose that prolonged oxidative stress in SCI rats leads to excessive nuclear translocation of HIF‐1α, contributing to upregulated HO‐1 expression and ferroptosis. HBO treatment likely mitigates this process by restoring redox balance and suppressing aberrant HIF‐1α signaling. This interpretation aligns with our proteomics data, which identified ferroptosis, glutathione metabolism, and the HIF‐1 signaling pathway as key mechanisms modulated by HBO.

Nevertheless, since HBO itself is known to activate Nrf2 and may also elevate HO‐1 expression under certain contexts [60], the overall change in HO‐1 levels observed in our study may have been masked by the dual regulatory effects. This possibility warrants further investigation in future studies.

In conclusion, our findings indicate that HBO attenuates oxidative damage and suppresses ferroptosis in the hippocampus of SCI rats, at least in part, through the activation of the Nrf2/GPX4 signaling pathway.

### 3.6 Nrf2 Inhibition Abolished the Antidepressant and Cognition‐Enhancing Effects of HBO

**FIGURE 6:**
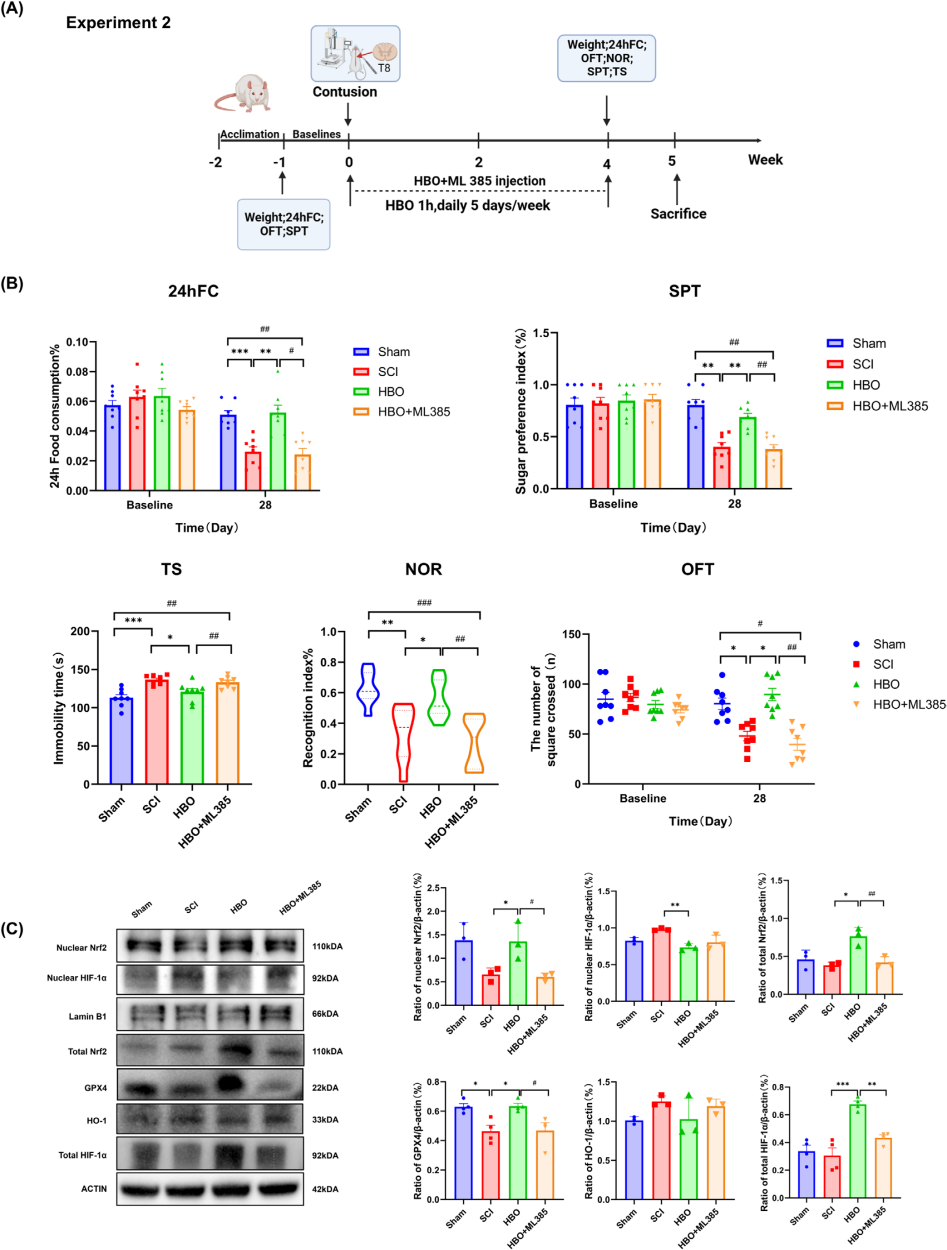
Blockade of Nrf2 abolished the effects of HBO on depressive behavior and cognitive impairment of SCI rats. (A) Timeline of Experiment 2. (B) Behavioral assessment of depressive‐like behavior and cognitive performance following coadministration of the Nrf2 inhibitor ML385 with HBO treatment in SCI rats. (C) Representative western blot bands and quantification of hippocampal protein expression levels of nuclear Nrf2, nuclear HIF‐1α, total Nrf2, HO‐1, total HIF‐1α, and GPX4. Data are presented as mean ± SEM, *n* = 4–8 per group, *p < 0.05, **p < 0.01, ***p < 0.001, ****p < 0.0001 versus SCI group, ^#^ *p* < 0.05, ^##^ *p* < 0.01, ^###^p < 0.001 versus HBO + ML385 group. Statistical analysis was conducted using two‐way ANOVA followed by Tukey's post hoc test.

To further delineate the role of Nrf2 in mediating the antidepressant and cognition‐enhancing effects of HBO treatment, SCI rats were administered ML385 (a selective Nrf2 inhibitor) 30 min prior to each daily HBO session (Figure 6A). Compared with SCI controls, HBO treatment significantly increased 24‐h food intake, sucrose preference, and exploratory behavior in the open field test, while reducing immobility time in the tail suspension test and improving performance in the novel object recognition task. These behavioral improvements were markedly abolished by co‐administration of ML385 (Figure 6B), implicating Nrf2 as a key mediator of HBO's neurobehavioral effects.

To confirm the involvement of Nrf2 signaling at the molecular level, we assessed hippocampal protein expression of nuclear and total Nrf2, nuclear and total HIF‐1α, HO‐1, and GPX4 by western blotting (Figure 6C). HBO treatment significantly upregulated nuclear and total Nrf2, total HIF‐1α, and GPX4 levels, while concurrently decreasing nuclear HIF‐1α expression. Notably, ML385 co‐treatment reversed these changes (substantially reducing Nrf2 and GPX4 expression as well as total HIF‐1α levels), thereby negating the molecular correlates of HBO's protective effects. However, nuclear HIF‐1α expression remained largely unaffected by ML385, and HO‐1 levels did not exhibit significant changes across groups.

These findings suggest that HBO exerts its antidepressant and cognitive benefits primarily through activation of the Nrf2/GPX4 signaling axis. Pharmacological inhibition of Nrf2 disrupts this pathway, eliminating HBO‐induced neurobehavioral and molecular improvements. Although we did not examine the effects of HBO in sham‐operated animals or the impact of ML385 alone in SCI rats, our results likely reflect the net effect of two opposing mechanisms (HBO‐induced activation and ML385‐induced inhibition of Nrf2 signaling).

Furthermore, the exact role of the HIF‐1α/HO‐1 axis in this context remains unclear. Given the complexity of HIF‐1α's regulatory role in oxidative stress and ferroptosis, additional studies are warranted to explore its interplay with Nrf2/GPX4 signaling in HBO‐mediated neuroprotection.

## 4 Conclusion

In summary, our study demonstrates that HBO treatment effectively alleviates depressive‐like behavior, cognitive impairment, neuroinflammation, and oxidative stress induced by SCI. Mechanistically, these beneficial effects are likely mediated through the suppression of ferroptosis via activation of the Nrf2/GPX4 signaling pathway. These findings provide new insights into the pathogenesis of SCI‐related neuropsychiatric deficits and highlight HBO as a promising noninvasive therapeutic strategy for preventing and mitigating depression and cognitive decline in patients with SCI.

## Author Contributions

C.L., F.L., X.L. and P.C.: conception and design. C.L., Z.W., C.D., M.S. and S.Y.: acquisition of data. C.L., P.C., X.L. and F.L.: analysis and interpretation of data. C.L. and F.L.: drafting the article. P.C., X.L. and F.L.: critically revising the article. F.L.: reviewed submitted version of manuscript. C.L. and X.L.: statistical analysis. X.L. and P.C.: administrative/technical/material support. F.L., P.C. and X.L.: study supervision.

## Consent

All authors read and approved thse final manuscript.

## Conflicts of Interest

The authors declare no Conflicts of Interest.

## Supporting information


Appendix S1.

## Data Availability

The data that support the findings of this study are available from the corresponding author upon reasonable request.
