# Supplementary material for: Regulation of Nrf2/GPX4 Signaling Pathway by Hyperbaric Oxygen Protects Against Depressive Behavior and Cognitive Impairment in a Spinal Cord Injury Rat Model
Source: CNS Neurosci Ther. 2025 May 6;31(5):e70421. doi: 10.1111/cns.70421 (PMC12053091; doi:10.1111/cns.70421)
Supplement: Supplementary file 1 — Appendix S1. [file CNS-31-e70421-s001.docx]

**Supplementary Material**

**Table of Contents**

1. Appendix Detailed procedures and information of Sucrose preference test, Morris water maze and DIA Quantitative Proteomic Analysis

2.Figures and legends of Cognitive impairment and depressive-like behavioral tests results, the relative expression level of ferroptosis related protein abundance of three groups and experimental proteomics procedure for proteomics of SCI and HBO groups.

**Appendix**

**Method**

**1.1** **Sucrose preference test**

Rats were caged and given a water bottle containing 1% sucrose solution (wt/vol) and a bottle with tap water. Each location averaged 30 g of food. After 48 h of adaptation, rats were handled for 30 min and returned to their cages for 24 h baseline measurement. To avoid location preference, the two bottles were switched every 12 h. Rats were fasted for 24 hours before the preference test. All rats were tested 12 h following deprivation. Before and after testing, water bottles, food, and rats were weighed. The sugar preference index (SPI) was derived by dividing sweetened water consumption by water consumption (sweetened and ordinary)×100%.

**1.2 Novel object recognition**

Twenty-four hours after the open field test, the rats were placed back in the same environment containing two similar sample objects (A + A). After a retention interval (1 h), the animals were returned to the test phase, in which the rats would encounter two different objects, one being the familiar and the other being novel (A + B). The time spent on exploring the objects was recorded by the video tracking software. The recognition index (B/ (A + B) × 100%) was the quantity of time spent exploring the novel object (B) in the test phase over the entire time spent exploring the objects in the test phase. A preference index above 50% indicated novel object preference, below 50% familiar object preference, and 50% no preference

**1.****3 Morris water maze**

*Trend pattern*：The water entry point of the experimental animal and the center line of the platform were taken as the central axis. If the distance between all the movement tracks of the animal and the central axis did not exceed 50% of the radius, and the movement time in this area was at least 70% of the total movement time, the movement of the experimental animal was considered to be a trend strategy.

Edge pattern：A circle of 75% of the pool radius was selected. If more than 70% of the time the animal moved outside the circle, the movement of the experimental animal was considered an edge strategy.

Random pattern：Unlike the above, the animal searched the entire pool without spatial bias.

**1.4. Procedures and information of DIA Quantitative Proteomic Analysis**

**1.4.1 Fractionation for DDA library Generation**

The MP FastPrep-24 homogenizer was used to initially homogenize the samples (24x2, 6.0 M/S, 60 s, twice), and SDT buffer (4% SDS, 100 mM Tris-HCl, pH 7.6) was then added. The BCA Protein Assay Kit (Bio-Rad, USA) was used to measure the protein concentrations after the samples were boiled and centrifuged. 20 µg of protein for each sample were mixed with 5X loading buffer respectively and boiled for 5 min. The proteins were separated on 4%-20% SDS-PAGE gel (constant voltage 180V, 45 min). Protein bands were visualized by Coomassie Blue R-250 staining.

For the purpose of creating a DDA library and ensuring its quality, an equal aliquot from each sample used in the experiment was combined into one sample.

**1.4.2. Protein digestion and Data Dependent Acquisition (DDA) Mass Spectrometry Assay and Mass Spectrometry Assay for Data Independent Acquisition (DIA)**

Each sample was mixed at 600 rpm for 1.5 h (37°C) with 10 mM DTT detergent. After cooling to room temperature, IAA was added at 20 mM to inhibit reduced cysteine residues and incubated for 30 min in darkness. The samples were then transferred to filters (Microcon units 10 kDa). The filters were washed three times in 100 μl UA buffer and twice in 25mM NH4HCO3 buffer. Finally, trypsin (1:50) was added to the samples and incubated at 37°C for 15-18 h (overnight) to collect the resulting peptides as a filtrate. The peptides of each sample were desalted on C18 Cartridges (Empore^TM^ SPE Cartridges C18 (standard density), bed I.D. 7 mm, volume 3 ml, Sigma), concentrated by vacuum centrifugation, and reconstituted in 40 μl of 0.1% (v/v) formic acid. UV spectral density at 280 nm calculated peptide content. For DIA experiments, iRT (indexed retention time) calibration peptides were spiked into the sample. Digested pool peptides were then fractionated to 10 fraction using Thermo Scientific^TM^ Pierce^TM^ High pH Reversed-Phase Peptide Fractionation Kit. Each fraction was desalted on Empore^TM^ SPE Cartridges C18 (standard density), bed I.D. 7 mm, volume 3 ml, Sigma, and reconstituted in 40μl of 0.1% (v/v) formic acid. iRT-Kits (Biognosys) peptides were spiked before DDA analysis.

Thermo Scientific's Q-Exactive HF-X mass spectrometer and Easy-nLC 1200 chromatography system injected all DDA library fractions. A linear gradient of buffer B (84% acetonitrile in 0.1% formic acid) at 300 nl/min separated the peptide on a C18 Analytical Column (Thermo Scientific, ES802, 1.9 μm, 75 μm*20 cm). MS detection was positive ion, scan range 350-1800 m/z, MS1 scan resolution 60000 at 200 m/z, AGC target 1e6, maximum IT 50ms, dynamic exclusion 10.0s. 20 inclusion list-based ddMS2 scans followed each complete MS–SIM scan. Isolation window was 1.5m/z, MS2 scan resolution was 30000(@m/z 200), AGC goal was 1e5, maximum IT was 50ms, and normalised collision energy was 30 eV.

**1.3.3** **Mass Spectrometry Assay for Data Independent Acquisition (DIA)**

Q-Exactive HF-X mass spectrometers attached to Easy-nLC 1200 chromatography systems in data-independent acquisition (DIA) mode evaluated each sample's peptides. 44 DIA scans spanning 350–1800 m/z with the following settings: SIM full scan resolution was 120,000 at 200 m/z; AGC 3e6; maximum IT 30ms; profile mode; DIA scans were 30,000; AGC target 3e6; Max IT auto; MS2 Activation Type HCD; normalised collision energy 30 eV.

**1.3.4.** **Mass spectrometry data analysis**

Spectronaut^TM^ 14.4.200727.47784 (Biognosys) scanned the FASTA sequence database for DDA library data. http://www.uniprot.org provided the database. Biognosys (iRT Kit) added iRT peptides. The parameters were trypsin, max missed cleavages 1, carbamidomethyl(C), oxidation(M), and acetyl (Protein N-term). All protein identification results were based on 99% confidence and FDR < 1%. Spectronaut^TM^ 14.4.200727.47784 searched the above spectrum library for DIA data. The main software parameters are dynamic iRT retention time prediction, MS2 level correction interference, and cross run normalisation. Q value threshold 0.01 (FDR<1%) filtered all results.

**1.3.5. Bioinformatics analysis**

Pareto-scaled Principal Component Analysis (PCA) was performed with the list of non-redundant proteins detected in at least three samples per group. Hierarchical clustering was performed on the differential proteins (DEPs) between groups (with p< 0.05, |Fold Change| > 1.2). Gene Ontology, enriched Disease Ontology, and Kyoto Encyclopedia of Genes and Genomes analyses were used to predict the functions of differentially expressed peptides and their precursor proteins. A two-tailed Fisher’s exact test was used to test the enrichment of the DEPs against all identified proteins. The top 20 were shown for annotation. The vertical axis displayed the categorization of function, pathway, and illness, and the horizontal axis displayed the percent of enriched genes in relation to the total number of genes. The bubble size displayed the number of genes, and the color displayed the p-value of enrichment significance.

**2.1 Figure and legend**

**
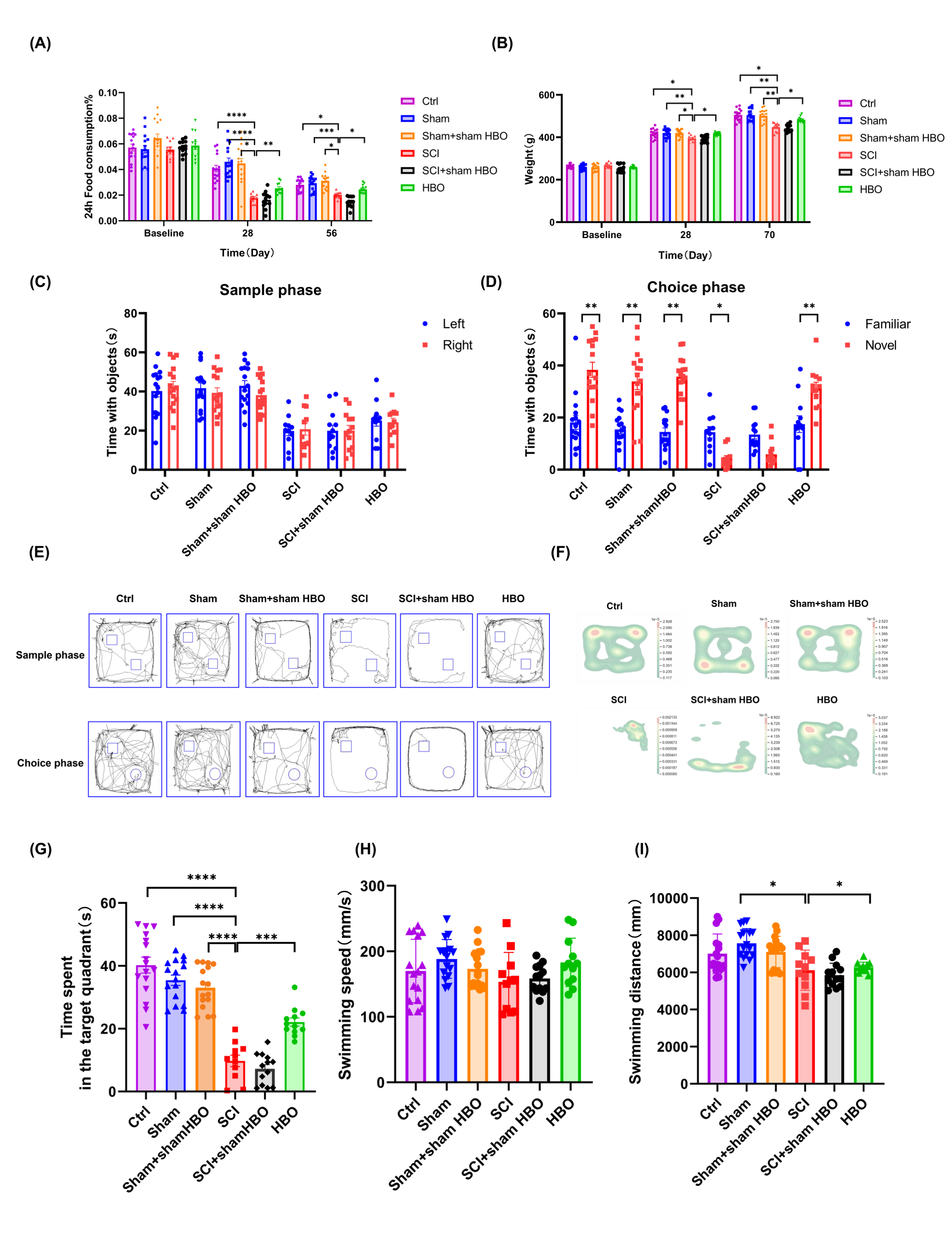
**

**Figure S1. Effects of HBO treatment on the prevention of neuropsychiatric deficits in SCI rats.**(A, B) 24-hour food intake and body weight gain among the Ctrl, Sham, Sham + sham HBO, SCI, SCI + sham HBO, and HBO groups.(C, D) Time spent in the sample and choice phases during the novel object recognition (NOR) test across all groups.(E) Representative trajectory plots from the NOR test.(F) Heatmap of open field exploration.(G) Time spent in the target quadrant during the probe trial of the Morris water maze (MWM).(H, I) Swimming speed and distance traveled during the MWM probe test.Data are presented as mean ± SEM (n = 11–16). Statistical significance was determined by mixed-effects model analysis with Tukey’s post hoc test, two-way repeated measures ANOVA followed by Bonferroni post hoc test, or one-way ANOVA with Tukey’s post hoc test.*p < 0.05, **p < 0.01, ***p < 0.001, ***p < 0.0001 vs. SCI group.


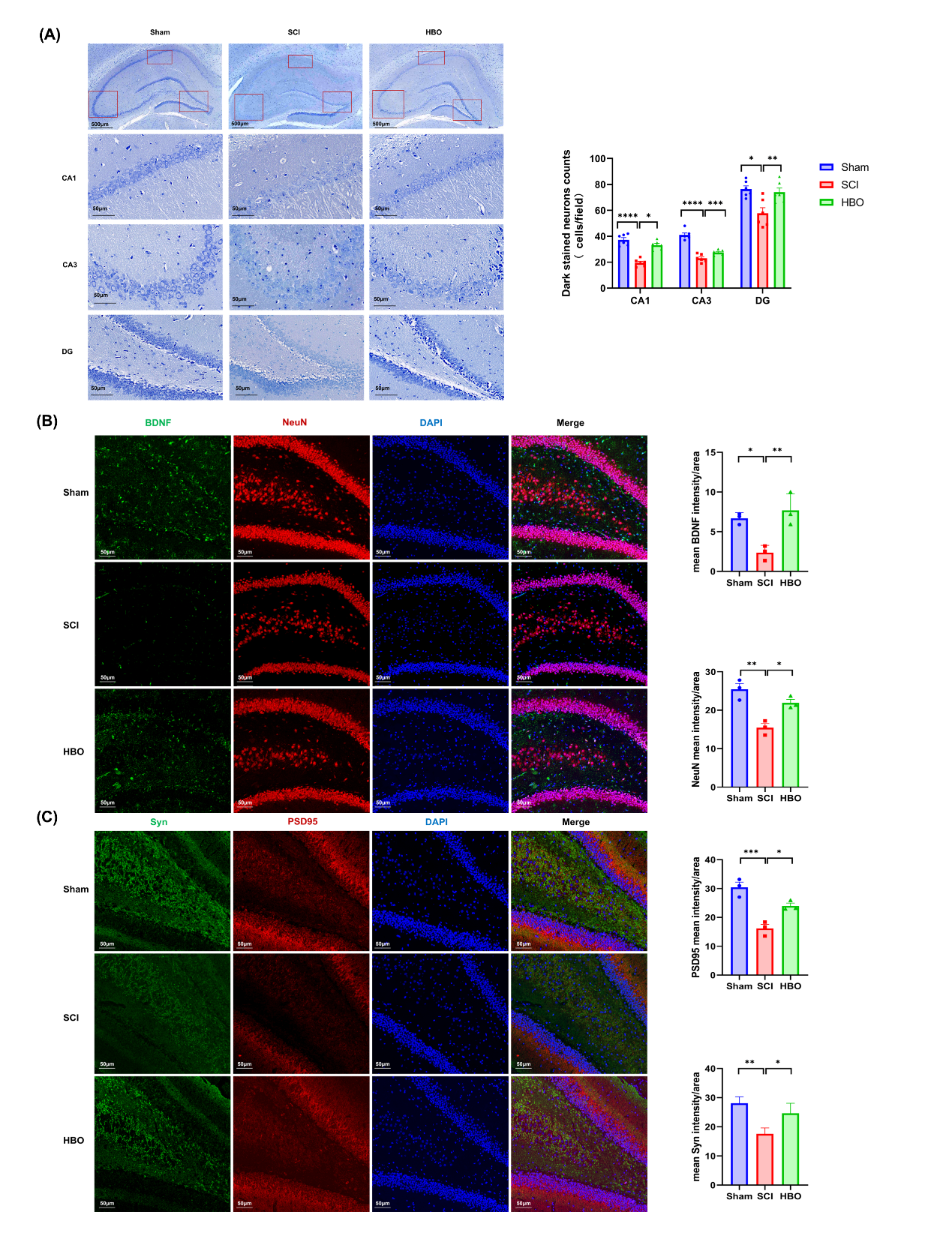


**Figure S2. HBO alleviates neuronal damage and promotes synaptic protein expression in the hippocampus.**
(A) Representative Nissl staining of hippocampal sections. Neurons in the SCI group were irregularly arranged and exhibited Nissl body dissolution compared to the Sham group. HBO treatment ameliorated this pathology. Red boxes indicate magnified regions. Scale bar = 500 μm; inset = 50 μm. CA: Cornu Ammonis; DG: dentate gyrus; DAPI: 4’,6-diamidino-2-phenylindole. Bar graph shows the number of dark-stained Nissl bodies.(B) Representative immunofluorescence staining of BDNF and NeuN co-localization in the hippocampus. Scale bar = 50 μm. Quantification of BDNF and NeuN fluorescence intensity is shown.(C) Representative immunofluorescence staining of PSD95 and Syn co-localization. Scale bar = 50 μm. Quantification of PSD95 and Syn fluorescence is shown.Data are presented as mean ± SEM (n = 3–6 per group). Statistical significance was determined by one-way ANOVA followed by Tukey’s post hoc test.*p < 0.05, **p < 0.01, ***p < 0.001, ****p < 0.0001 vs. SCI group.*


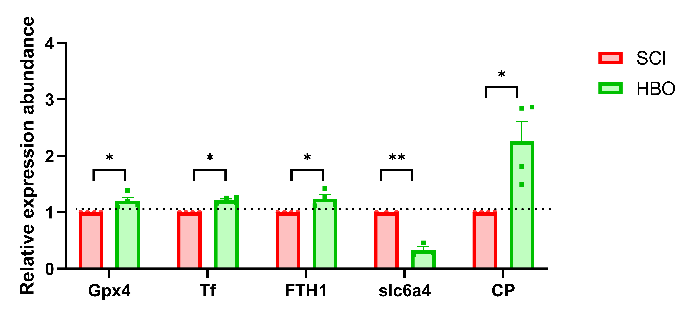


**Figure S3. Relative expression levels of ferroptosis-related proteins among the three groups.** Protein levels were normalized and expressed as fold changes relative to the SCI group. Data are shown as mean ± SEM.*p < 0.05, **p < 0.01 vs. SCI group.*


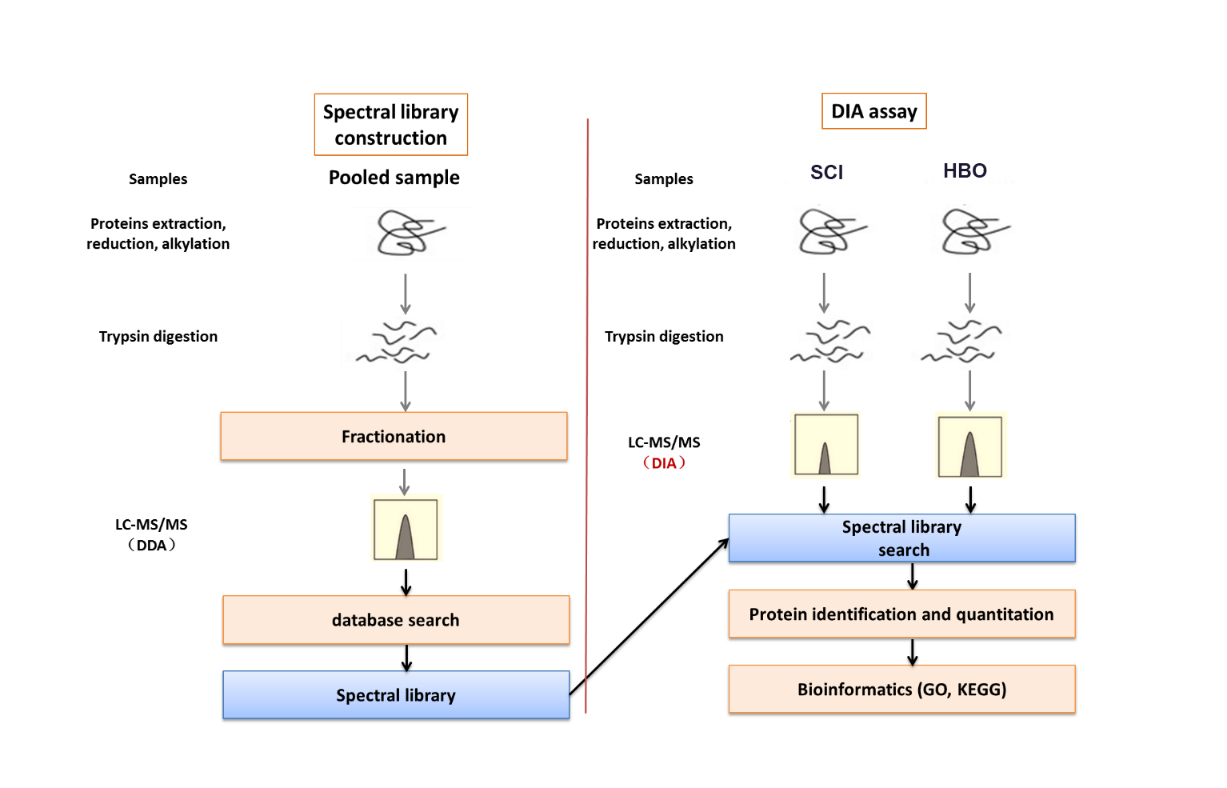


**Figure S4. Workflow of hippocampal proteomics in SCI and HBO groups.** Schematic overview of sample preparation, data-independent acquisition (DIA) proteomic workflow, and bioinformatics pipeline.
